# Epigenetic Diversity and the Evolutionary Potential of Wild Populations

**DOI:** 10.1111/eva.70011

**Published:** 2024-10-21

**Authors:** Miguel Baltazar‐Soares, Alice Balard, Melanie J. Heckwolf

**Affiliations:** ^1^ Department of Biology University of Turku Turku Finland; ^2^ School of Biological and Behavioural Sciences Queen Mary University of London London UK; ^3^ Leibniz Centre for Tropical Marine Research Bremen Germany; ^4^ Smithsonian Tropical Research Institute Gamboa Panama

**Keywords:** acclimatisation, adaptive potential, conservation biology, epigenetics, natural populations

## Abstract

Fast‐paced selective pressures imposed by climate change and anthropogenic activities call for adaptive evolutionary responses to emerge at ecological timescales. However, the evolution and heritability of genomic variation underlie mechanistic constraints, which dictate a slower pace of adaptation exclusively relying on standing genetic variation and novel mutations. Environmentally responsive epigenetic mechanisms can allow acclimatisation and adaptive phenotypes to arise faster than DNA sequence‐based mechanisms alone. Nevertheless, the knowledge gap between identifying epigenetic marks and effectively deeming them functional is still wide in a natural context and often outside the scope of model organisms. With this Special Issue, we aimed to narrow this gap by presenting a compilation of original research articles, reviews and opinions on the topic of epigenetics in wild populations. We contextualised this collection within the overarching topic of conservation biology, as we firmly propose that epigenetic research can significantly enhance the effectiveness of conservation measures. Contributions highlighted the putative role of epigenetic variation in the acclimatisation and adaptive potential of species and populations directly and indirectly affected by climatic shifts and anthropogenic actions. They further exemplified how epigenetic variation can be used as biomarkers for monitoring variations in physiology, phenology and behaviour. Lastly, reviews and perspective articles illustrated the past and present of epigenetic research in wild populations while suggesting future research avenues.

## 1 Introduction

The global biodiversity loss due to growing anthropogenic threats, such as habitat loss, pollution and climate change, calls for effective nature conservation and management strategies (Baltazar‐Soares, Brans, and Eizaguirre 2021; Sage 2020). Integrating evolutionary and ecological perspectives can significantly enhance the efficiency of conservation practices, often achieved with molecular tools (Bernatchez et al. 2024; Eizaguirre and Baltazar‐Soares 2014). In this context, DNA‐based molecular markers have become indispensable in the evaluation of evolutionary processes, as they allow for an effective assessment of species resilience with direct implications for practical conservation efforts (Hogg et al. 2022; Theissinger et al. 2023). Their applications range, for instance, from parentage analyses and connectivity monitoring to the use of environmental DNA for species detection (Deiner, Yamanaka, and Bernatchez 2021; Galla et al. 2020). However, the DNA carries more information than just DNA sequence variation: epigenetic variation offers an incredibly promising and thus far mostly untapped source of crucial insights for conservation.

Here, we define epigenetics as meiotically or mitotically heritable genomic information that can result in gene regulatory changes without requiring alterations to the DNA sequence. DNA methylation, histone modification and non‐coding RNAs are currently the most studied epigenetic marks. It was only during the last decade that epigenetic research expanded into non‐model organisms and wild populations (Rey et al. 2020). Since then, it has been demonstrated that phenotypic expression can be modulated by factors beyond the coding DNA across a variety of traits and species.

This Special Issue originated from a symposium held at the 2022 European Society of Evolutionary Biology (ESEB) meeting in Prague, Czech Republic. Our goal was to compile robust examples of epigenetic research on non‐model organisms, under the theme ‘Epigenetics goes wild! Epigenetic diversity and the evolutionary potential of wild populations’. As our knowledge of molecular mechanisms of inheritance and adaptation grows, so should our toolbox of molecular markers, both in quality and quantity. The premise is that if our goal is to understand natural processes in all their complexity, this goal should be met with comprehensive tools, hypotheses and analyses that can capture the different aspects of this intricacy.

Showcasing examples of such tools and analyses, this Issue covers three main aspects: (1) the involvement of epigenetics in *adaptive processes*, (2) the role of epigenetics as *biomarkers* for monitoring physiological and behavioural processes, and (3) the value of *comparative epigenetics* in validating patterns. Collectively, these topics offer current conservation and monitoring tools while also providing a glimpse into the future of epigenetic research in wild populations, potentially paving the way for a distinct line of research in conservation epigenetics.

## 2 Epigenetic Changes Promote Acclimatisation and Adaptive Potential in the Wild

Epigenetic marks are a known source of phenotypic diversity through which they can contribute to rapid evolution (Ashe, Colot, and Oldroyd 2021). On the one hand, epigenetic marks that directly respond to the environment promote an organism's acclimatisation potential, here defined as the ability to modify the phenotype in response to environmental changes without altering the genotype. On the other hand, spontaneous epimutations that remain environmentally stable across generations can reflect selective pressures similar to DNA‐based mutations under selection. Therefore, they can contribute to the emergence of adaptive phenotypes at a faster pace, as they occur three to five orders of magnitude more frequently than DNA‐based mutations (Van Der Graaf et al. 2015).

Understanding the acclimatisation and adaptation potential of reef‐building corals is a pressing conservation issue, since coral reefs are particularly vulnerable to global warming, with a devastating decline of 70%–90% at 1.5°C global air temperature increase (IPCC 2018). By investigating the DNA methylation and expression plasticity in a thermal acclimation experiment in a reef‐building coral (*Acropora nana*), Guerrero and Bay (2024) were able to pinpoint specific DNA methylation shifts that are associated with thermal conditions and acute heat stress. Similarly, Abbott, Loockerman, and Matz (2024) also exposed another reef‐building coral (*Acropora millepora*) to increased water temperatures simulating a natural heat wave with subsequent recovery under control temperature. Interestingly, two types of molecular responses were observed: heat‐induced genes that did not revert expression back to base level after recovery and heat‐induced genes that changed the magnitude of expression following temperature fluctuation. Similar to the previous study, Abbott and colleagues showed an association between gene body methylation and environmental conditions. While DNA methylation changes in both studies were not directly correlated with expression changes, leaving a question mark as to their specific phenotypic implications, they could serve as biomarkers to infer past heat waves in natural reefs and thus serve an important monitoring purpose.

Another important ecosystem builder is the kelp *Saccharina latissima*, a macroalgae with both economical (as saccharine producer) and ecological (marine ecosystem builders) significance. Contrary to land plants, kelps do possess methylated chloroplasts and thus another potential mechanism to overcome climatic shifts. Scheschonk et al. (2024) are the first to report the chloroplast methylome of *S. latissima*, which holds a specific signature for each location of origin. To infer whether this signature was population‐specific or a response to the local environment, the authors exposed kelp to different temperature regimes. Despite temperature regimes not triggering substantial differences in methylation patterns of the chloroplasts, authors reported a genome‐wide effect in terms of methylation level and methylated sites when assessing population‐specific environments—suggestive of local adaptation. Indeed, inferring signatures of local adaptation and population structure benefits from both a genomic and epigenomic approach, as Chain et al. (2024) showed through complementary variation in copy number (CNV) and DNA methylation of stickleback (*Gasterosteus aculeatus*) populations across a salinity range.

A whole other set of issues due to global warming is faced by ectothermic species. Analysing the effects of warming on the fitness of loggerhead turtle hatchlings (*Caretta caretta*) beyond the pure sex‐ratio shift, Yen et al. (2024) showed that an increased incubation temperature has sublethal effects, such as weaker locomotion and a lower dispersal capacity, affecting their fitness after egg emergence. They identified genomic sites with an altered DNA methylation between temperature treatments. Their results suggest that the methylome can carry signatures of sub‐lethal effects under global warming conditions. The strong evidence regarding the relationship between fitness‐related traits and methylation shows the biomarker potential of epigenetics, which is expanded in the section below.

## 3 Physiology, Phenology, Behaviour and More: Epigenetics as Biomarkers of Fitness‐Related Processes

Monitoring strategies are critical to the success of conservation measures, making the development of efficient, cheap and accurate molecular monitoring tools an invaluable first step in any conservation project. While the development of epigenetic biomarkers is still in its infancy, the range of studies on the biological processes they potentially represent continues to expand. Balard et al. (2024) summarised examples of DNA methylation‐based biomarkers that have been used to determine an individual's age, health status or sex, allowing conclusions on a population's reproductive capacity and resilience. Importantly, age determination based on DNA methylation sites is a process that can be standardised across taxa, as shown by Heydenrych et al. (2024). They proposed a machine learning‐based model to identify CpG‐dense gene promoter regions, which can predict age at sexual maturity across sexes and taxa. Since CpG sites are the main targets for DNA methylation in vertebrates, this approach reflects an elegant indirect way of using epigenetic information. The establishment of a cross‐species epigenetic biomarker, currently emerging in ageing research, presents an encouraging model for identifying other conserved biological indicators across different organisms. Beyond ageing, epigenetic patterns are correlated with fitness‐related traits, paving the way for further biomarker development. By exploring mechanisms associated with lay date in great tits (*Parus major*), Lindner et al. (2024) provided evidence that genetic and epigenetic differentiation between early and late laying selection lines affected complementary genomic pathways. In their experiment, DNA methylation was hypothesised to act downstream of neuroendocrine pathways influencing reproductive timing. Also, on great tits, Sepers, Verhoeven, and van Oers (2024) performed a cross‐fostered experiment to test whether lasting early developmental phenotypic changes are associated with post‐developmental DNA methylation changes. They concluded that DNA methylation changes were associated with nutritional stress during early development (pre‐fledging) through indirect carry‐over effects, thus potentially impacting the fitness of early life stages. This suggests that great tits early life stages are rich in epigenetic information that is indicative of fitness in adults. Beyond these developmental predictions, epigenetic patterns have also been shown to carry information on the experiences of the parental generation: Hukkanen et al. (2023) showed that elevated prenatal glucocorticoids in great tits result in CpG site‐specific DNA methylation changes in the glucocorticoid receptor gene, suggesting that maternal hormones can influence their offspring stress response through epigenetic mechanisms. Considering the fitness value of life history events revolving around reproduction in birds, that is, laying timing, reproduction timing and hatching date, the studies by Sepers et al., Lindner et al., and Hukkanen et al. are certainly contributing to the foundations of an avian‐targeted epigenetic biomarker set predictive of adult fitness.

In mammals, fertility was correlated with epigenetic patterns, which can be a particularly important tool in cases of extremely rare species whose population's viability should be monitored non‐invasively (Tennenbaum et al. 2024). In their article, Tennenbaum et al. (2024) reported DNA methylation changes in gene pathways linked to male fertility in the endangered black‐footed ferret (*Mustela nigripes*) managed ex situ. In a rich review, Villalba de la Peña and Kronholm (2024) have elaborated on how antimicrobial resistance can be traced with epigenetic marks as some mechanisms—such as reversible phenotypes and hetero‐resistance—are argued to have an epigenetic basis. Their excellent work further presents cutting‐edge epigenetic editing techniques that could in the future be applied to antimicrobial hamper resistance in the wild.

## 4 Comparative Epigenomics and Potential Future Applications in Conservation

Comparative genomics enables us to understand how generalisable evolutionary patterns are across species, bringing to light different molecular solutions to tackle similar evolutionary pressures. Extending the concept to comparative epigenetics will aid our understanding not only of how common specific epigenetic responses to environmental challenges are, but also lays the foundation to develop multi‐species biomarkers. In a holistic approach to molecular adaptation, Wang et al. (2024) screened for DNA methylation, transcriptomic and genomic variation among three macaque species to investigate the machinery behind distinct growth patterns and habitat preferences. The authors identified cross‐species differentially methylated regions (DMR) in candidate genes putatively associated with the aforementioned traits. In addition, some DMR genes also showed differential expression suggestive of the gene regulatory potential of DNA methylation. Beyond that, DNA methylation patterns can also be indicative of diet. Chen et al. (2024) identified patterns of epigenetic regulation among polar bears, giant pandas and red pandas to investigate diet‐driven adaptive evolution. In conclusion, in a comparative assessment of methylomes, Çokoğlu et al. (2024) brought an interesting scope on human evolution and archaeogenomics by trying to retrieve signatures of sex, tissue source and lifestyle transition from hunter‐gatherers to farmers in the DNA methylation of palaeogenomes. They found that the lab of origin was the main driver and provided guidelines to reduce this technical noise in the future. Arguably, characterising patterns across taxonomic borders will allow tool standardisation, thus enhancing the cost‐ and time‐efficiency of marker development and the sustainability of applications.

From the point of view of epigenetics in the wild, however, there is a lot more to be explored. On the one hand, the extensive review of Baduel et al. (2024) is a great starting point for those interested in learning about the link between genetic and epigenetic patterns and uses published studies to synthesise our current level of understanding on the interplay that shapes phenotypic variation. On the other hand, Balard et al. (2024) elaborated on how epigenetic tools can become complementary to traditionally used molecular tools in conservation by pinpointing specific examples and offering novel frameworks to do so. After introducing commonly used epigenetic screening strategies, they also propose novel approaches, such as epi‐eDNA and epigenetic‐based ecological modelling, to match current technological advances in molecular‐based research.

In conclusion, we can first and foremost safely state that providing conclusive evidence of the functionality of epigenetic marks is one of the holy grails in evolutionary biology. We acknowledge that despite many aspects remaining elusive—such as the advocated link between DNA methylation and gene expression regulation—the quality of research presented here significantly reduces such current knowledge gaps. We see this collection of articles—together with many others that have been published recently (Hu and Barrett 2017; Husby 2022; Rey et al. 2020)—as a major contribution to the overarching goal of ensuring a comprehensive understanding of species' adaptive potential.

## Conflicts of Interest

Miguel Baltazar‐Soares, Alice Balard and Melanie J. Heckwolf are Editorial Board members of Evolutionary Applications and co‐authors of this article. To minimise bias, they were excluded from all editorial decision‐making related to the acceptance of this article for publication.

## Data Availability

The authors have nothing to report.
